# microRNA expression patterns across seven cancers are highly correlated and dominated by evolutionarily ancient families

**DOI:** 10.3892/br.2014.239

**Published:** 2014-02-17

**Authors:** ERIC J. DEVOR, BRANDON M. SCHICKLING, KIMBERLY K. LESLIE

**Affiliations:** 1 Department of Obstetrics and Gynecology, University of Iowa Carver College of Medicine, Iowa City, IA 52242, USA; 2 Department of Internal Medicine, University of Iowa Carver College of Medicine, Iowa City, IA 52242, USA; 3 Holden Comprehensive Cancer Center, University of Iowa Hospitals and Clinics, Iowa City, IA 52242, USA

**Keywords:** microRNA, The Cancer Genome Atlas, rank correlation

## Abstract

microRNAs (miRNAs) are involved in almost all normal and pathogenic eukaryotic cell processes. One area in which the influence of miRNAs is most prominent is cancer. Numerous expression surveys and more focused studies have revealed miRNA involvement in carcinogenesis, cellular pathology, cell behavior and prognosis. Large-scale comparisons of miRNA expression in varioius types of cancer have not been previously possible. However, The Cancer Genome Atlas (TCGA), an extensive multi-centered effort to characterize the genomes of hundreds of types of cancer, has enabled such comparisons. In the present study, the expression patterns of hundreds of miRNAs in thousands of tumors covering seven types of cancer: uterine corpus adenocarcinoma, ovarian serous adenocarcinoma, breast adenocarcinoma, prostate adenocarcinoma, pancreatic adenocarcinoma, colorectal adenocarcinoma, and lung adenocarcinoma were analyzed. The results showed that miRNA expression patterns among these cancer types are highly correlated (0.874>ρ>0.974) and that miRNA expression in all seven cancer types is dominated by miRNAs belonging to the most evolutionarily ancient miRNA families. This raises the possibility that more ancient miRNAs are involved in the fundamental cell processes that are central to tumor evolution.

## Introduction

Following the completion of the first human genome sequence, the National Institutes of Health launched a substantial effort to sequence the genomes of a number of cancers in hundreds of patients (1). Over the past few years these data and the genomic inferences gained from them, known collectively as The Cancer Genome Atlas (TCGA), have begun to appear in publically accessible form. These releases have been accompanied by a very important series of data analyses under the auspices of The Cancer Genome Atlas Research Network (www.nature.com/ng/focus/tcga/index.html) (2–4). Among the information released is the expression of up to 1,046 miRNAs in thousands of tumors. Given the stringency governing the acquisition and preparation of materials in the TCGA, both large- and small-scale global analyses of data within and between various types of cancer can be carried out with confidence previously unattainable.

In the present study, we extracted miRNA expression data on seven types of cancer for the purpose of examining shared and unique expression patterns. The cancer types we selected to extract are uterine corpus adenocarcinoma (designated as ENDOCA), ovarian serous adenocarcinoma (OVARCA), breast adenocarcinoma (BRSTCA), prostate adenocarcinoma (PROSCA), pancreatic adenocarcinoma (PANCCA), colorectal adenocarcinoma (COLNCA), and lung adenocarcinoma (LUNGCA). Our rank order correlation analyses of miRNA expression in these seven types of cancer reveal a high degree of homogeneity. Closer examination of individual miRNAs shows that evolutionarily ancient miRNA families are significantly over-represented among the most highly expressed miRNAs. We hypothesized that the two observations suggest that relatively minor variations in miRNA expression are sufficient to play a role in establishing and maintaining tumorigenesis and the regulatory targets of many of the most highly expressed miRNAs are likely to be involved in fundamental cellular processes that are re-programmed in cancer.

## Materials and methods

The data used in this study and the analyses reported herein have been approved by the The Cancer Genome Atlas Program Office at the National Cancer Institute of the United States National Institutes of Health (25/10/13).

TCGA miRNA expression in each cancer was determined via deep sequencing tumor-derived RNAs on the IlluminaHiSeq_miRNASeq platform (?). Potential microRNA sequences were verified against primary miRNA (pri-miRNA) and precursor miRNA (pre-miRNA) sequences contained in the miRBase microRNA database. Molecule counts for human miRNA sequences were standardized within each miRNA and reported as ‘hits’ per megabase (10^6^ bases). Standardization was performed to avoid bias potentially introduced by closely related miRNAs or by miRNAs occurring in clusters.

Standardized miRNA hit data for 1,046 miRNAs in 415 uterine corpus tumors (designated as ENDOCA), 770 breast tumors (BRSTCA), 286 prostate tumors (PROSCA), 62 pancreatic tumors (PANCCA), 478 colorectal tumors (COLNCA), and 482 lung tumors (LUNGCA) were extracted. However, TCGA reported expression data on only 680 miRNAs in 970 ovarian tumors (OVARCA). We therefore generated two separate files, one without OVARCA containing 1,046 miRNAs for each tumor and one with OVARCA that was trimmed to contain only 680 OVARCA miRNAs. In all of the miRNA hit data we calculated the average number of hits per miRNA per cancer and then re-ordered them. We compared miRNA expression in all seven cancer types pairwise (21 total comparisons) using the Spearman Rank Order Correlation (ρ) (5) (www.wessa.net/rankcorr.wasp). Statistical significance was assessed by the t-test with (n−2) degrees of freedom.

Regulatory targets of miRNAs were predicted via a number of different algorithms. Predicted target mRNA lists were compiled from three sources: TargetScan (targetscan.org), PicTar (pictar.mdc-berlin.de) and miRanda (microRNA.org). Targets appearing on at least two of the three sources were compiled for specific miRNAs and then supplemented with experimentally confirmed targets listed in TarBase (diana.imis.athena-innovation.gr). The mRNAs were then submitted for pathway analysis using The Database for Annotation, Visualization and Integrated Discovery (DAVID) version 6.7 online tool (6,7).

## Results

**Figure 1:**
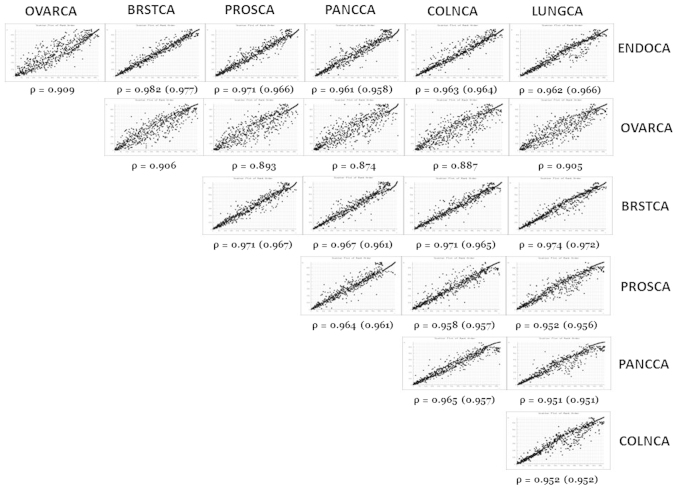
Rank order scatter plots of miRNA expression among the seven types of cancer. The Spearman rank order correlation coefficients based on 680 miRNAs and on 1,046 miRNAs (in parentheses) are also shown. Correlations are statistically significant (p<0.001). ENDOCA, uterine corpus adenocarcinoma; OVARCA, ovarian serous adenocarcinoma; BRSTCA, breast adenocarcinoma; PROSCA, prostate adenocarcinoma; PANCCA, pancreatic adenocarcinoma; COLNCA, colorectal adenocarcinoma; LUNGCA, lung adenocarcinoma.

Pairwise rank order scatter plots of miRNA expression among the seven types of cancer are shown in Fig. 1. These scatter plots indicate that there is great similarity in miRNA expression ranks among all of the cancers. This high degree of similarity is confirmed in the Spearman Rank Order Correlations (ρ) (Fig. 1). Pairwise rank order correlations are highly significant (p<0.0001), however, ovarian tumors consistently exhibit the lowest correlations (mean ρ=0.896±0.014) while breast tumors exhibit the highest correlations (mean ρ=0.961±0.028). Scatter plots and correlations shown in Fig. 1 were determined using trimmed (n=680) miRNA rank data, i.e., expression data for the six types of cancer reporting 1,046 miRNAs were trimmed to match the 680 miRNAs reported for ovarian cancer, after which the miRNAs were re-ordered.

The Spearman Rank Order Correlations (ρ) among the six types of cancer for which ranks were assigned on the basis of 1,046 miRNAs are also shown in Fig. 1. These correlations are almost identical to the trimmed correlations. Thus, no bias was introduced by trimming the miRNA ranks due to the fact that almost none of the 366 miRNAs that were not included in the ovarian cancer data exhibit any expression (mean hits ≤1.0) in the other six cancers.

## Discussion

In the current study, we have presented a simple rank correlation analysis of miRNA expression in seven types of cancer: uterine, ovarian, breast, prostate, pancreas, colorectal and lung. Our results show that all seven cancer types exhibit significantly correlated miRNA expression patterns. Although highly significant, the least similar of the expression patterns was observed in comparisons with ovarian tumors, likely due to the fact that, among the seven cancers, only ovarian tumors are uniformly of the serous histologic type, which are known to be more aggressive with a less favorable prognosis than the adenocarcinomas typical of the other six cancers used in this study. Thus, histologic similarity is potentially an aspect of the higher average miRNA expression rank correlations among the other six cancers.

The initial impetus for analyzing TCGA miRNA expression patterns originated from a previous study of miRNA expression in endometrial adenocarcinoma (8). The aim of the study was to compare the rank order correlation between our miRNA expression data, based on qPCR miRNA arrays of eight tumors, with that of the TCGA, based upon deep sequencing of 415 tumors. After censoring the two data sets using human miRNA sequences archived in miRBase Release 20 (miRBase.org), the identity of 276 miRNAs was confirmed. An expression rank order correlation showed that the two data sets were in significant agreement (ρ=0.718, p<0.0001, df=274). Thus, results obtained from our small miRNA study were entirely consistent with those obtained in a large-scale study utilizing a completely different technology. In a subsequent meta-analysis of miRNA expression studies of endometrial cancers (9), we noted that many of the miRNAs whose expression levels were reported to be significantly different in endometrial adenocarcinomas compared with benign endometrial tissues were involved in cell processes including response to hypoxia, anti-apoptosis, the crucial epithelial to mesenchymal transition and other cancer phenotypes such as invasiveness and metastasis (10,11). In addition, we showed that these chronically dysregulated miRNAs were all members of evolutionarily ancient miRNA families (12–15). It was found in the TCGA endometrial cancer data that the same ancient miRNA families were significantly over-represented in the most highly ranked miRNAs.

**Figure 2:**
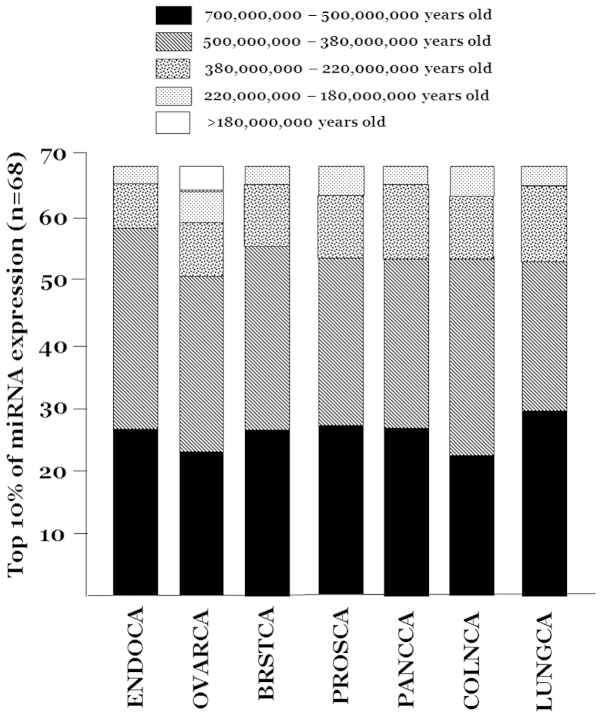
Evolutionary age composition of the miRNA families representing the top 10% of miRNA expression in the seven types of cancer (based on 680 miRNAs each). ENDOCA, uterine corpus adenocarcinoma; OVARCA, ovarian serous adenocarcinoma; BRSTCA, breast adenocarcinoma; PROSCA, prostate adenocarcinoma; PANCCA, pancreatic adenocarcinoma; COLNCA, colorectal adenocarcinoma; LUNGCA, lung adenocarcinoma.

Examination of miRNA expression ranks of the other six cancers showed that the same ancient miRNA families found to be significantly over-represented among the most highly expressed miRNAs in endometrial cancer were also significantly over-represented in the six types of cancer. There are 162 miRNAs belonging to families that first appeared in animal genomes >380,000,000 years ago, i.e., prior to the emergence of land animals (16). In all seven cancer types, the most ancient miRNAs comprise three quarters or more of the top 10% of miRNA expression (Fig. 2). Moreover, among the miRNAs in the top 10% <380,000,000 years, almost none of the miRNAs is a member of a family that post-dates the emergence of the Eutheria (mammals) 225,000,000 years ago (13,17).

Among the ‘younger’ miRNAs is the ubiquitous miR-21. This miRNA is ranked first or second in six of the seven cancer types examined in this study. miR-21 is well-known to play an important role in a wide range of normal and pathologic biological processes including development, inflammation, cardiovascular and pulmonary function and cancer (18). Among the more ancient miRNAs, miR-10 is the oldest known animal miRNA family (13). The two members of this family have average ranks of 9.9 (miR-10a; range, 3–27) and 8.1 (miR-10b; range, 1–25) among the seven types of cancer. The DAVID analysis of predicted miR-10 targets yielded a statistically significant enrichment of pathways related to cell differentiation. Similarly, miR-22, a single member family that first appeared among the bony fishes ~400,000,000 years ago, has an average rank of 5.7 (range, 2–20). Among the predicted mRNA targets of miR-22 are known cancer-related genes such as phosphatase and tensin homolog (PTEN), metadherin (MTDH), cyclin-dependent kinase 6 (CDK6), and laminin γ1 (LAMC1). Several cancer-related pathways and pathways related to the regulation of cell development are significantly enriched in the miR-22 DAVID analysis. Other miRNAs, including the miR-200 family (miR-141, miR-200a,b,c and miR-429) and miR-205, which emerged in animal genomes prior to the evolution of land animals, are involved in regulating crucial cancer-related functions, such as the epithelial to mesenchymal transition. This pattern is repeated throughout the most highly expressed and ancient miRNAs. We suggest that over-representation of these ancient miRNAs among the most highly expressed miRNAs in various cancers is simply a reflection of linkage between extremely ancient miRNAs and basic cell processes that are re-programmed in cancer.

The results have shown that miRNA expression among different human adenocarcinomas is significantly correlated. Moreover, the ranks of the most highly expressed miRNAs in these cancers are occupied by representatives of evolutionarily ancient miRNA families. This pattern is the result of ancient linkages between regulatory miRNAs and mRNA targets essential to carcinogenesis. Thus, it is among these most ancient miRNAs and their targets that attention should be focused. Recently, Hamilton *et al* (19) discovered an miRNA superfamily displaying pan-cancer oncogenic effects that are linked together by a conserved, shared core seed sequence motif. Members of this superfamily include miR-17, -18, -19, -93, -130, -210, and -455 seed families. None of these seed families is younger than 380,000,000 years.
